# Inhibition of SoxB2 or HDACs suppresses *Hydractinia* head regeneration by affecting blastema formation

**DOI:** 10.1080/19420889.2018.1450032

**Published:** 2018-04-03

**Authors:** Hakima Flici, Uri Frank

**Affiliations:** Centre for Chromosome Biology (CCB), School of Natural Sciences, National University of Ireland, Galway H91 CF50, Ireland

**Keywords:** blastema, cell proliferation, histone deacetylase, Hydractinia, regeneration, SoxB transcription factors

## Abstract

Regeneration has long been known to occur in the cnidarian *Hydractinia*. This process refers to its ability to regrow structures, i.e a head, lost by injury, a phenomenon that depends on the migration of proliferative cells to the site of injury, and the formation of a blastema, a mass of undifferentiated cells that will restore the missing head tissues. In our study, we showed that members of SoxB transcription factors and HDACs are involved in the regulation of *Hydractinia* neurogenesis in tissue homeostasis and regeneration. Particularly, we revealed that knockdown of SoxB2 or Hdac2 (a class I HDAC) knockdown, or inhibition of HDAC activity, suppress head regeneration. Here, we show that SoxB2 knockdown, or the inhibition of HDACs activity by TSA, a HDAC Class I and II inhibitor, interfere with head regeneration by affecting the migration of proliferative cells and the formation of a proliferative blastema.

One of the key strategies to advance our knowledge on regeneration is to study it in different animals and tissue contexts, as each system provides distinct perspectives for understanding the biology of this phenomenon. *Hydractinia*, a colony-forming animal, is capable of regenerating both distal and proximal structures: a polyp can regenerate a decapitated head and an isolated polyp can regenerate a colony [1,2,3]. Following decapitation and wound closure head regeneration proceeds with the recruitment of proliferative cells to the prospective head and formation of a blastema [1].

Based on their high-mobility group (HMG)-domain and functional properties, SoxB genes can be divided into two main subgroups, SoxB1 and SoxB2 [4, 5]. In mammals, SoxB1 proteins are transcriptional activators with an essential role in the maintenance of neural stem cells. SoxB2 proteins are transcriptional repressors, playing a role in neural stem cells differentiation [6]. Phylogenetic analyses showed that genomes of cnidarians, the sister group of Bilateria, encode at least three *SoxB*-like genes, but their affiliation with SoxB subgroups is not clear (Supplemental Files 1 and 2) [7,8,9].

Protein acetylation, the addition of an acetyl-group to lysine residues by an acetyltrasferase (HAT), is a modification that can be removed by a deacetylase (HDAC). HDACs are subdivided into four different classes and two different families. The classical HDAC family is composed of classes I, II and IV, that share sequence similarity within their catalytic domain and require Zn^2+^ ion as a cofactor. The sirtuin family contains members of class III HDACs, and do not share sequence homology with the members of the classical HDAC family. They use NAD^+^ as a cofactor [10,11]. Phylogenetic analysis indicated that the common eumetazoan ancestor had all HDAC subfamilies that were inherited by Cnidaria and Bilateria (Supplemental Files 3 and 4) [9].

Genomic deletion study in axolotl has revealed Sox2, a SoxB1 protein, to be important for spinal cord neural stem cell amplification during tail regeneration [12]. The conditional deletion of *Sox2* from the epithelium of the trachea in mice showed a reduced capacity to repair after injury [13]. In *Xenopus*, Sox2^+^ cells were shown to be important for tail and spinal cord regeneration [14,15]. During ear regeneration in zebrafish embryos, *Sox2*-deletetion prevented support cells from trasdifferentiation into hair cells [16]. Regarding the role of HDACs in animal regeneration, it was shown that the pharmacological inhibition of Class I/II HDACs inhibits tail and limb regeneration in *Xenopus* [17,18]. *Hydractinia* has three *SoxB* genes (*1*, *2* and *3*), six zinc-dependent *HDAC* genes (Class I (*2*, *3*, and *8*), Class II (*4* and *6*), and Class IV (*11*)), and six Sirtuin genes (Supplemental Files 1–4) [9]. In *Hydractinia* polyps, *SoxB2* and *Hdac 2*, *3* and *4* are largely expressed in the lower body part, a highly proliferative area, and the downregulation of *SoxB2* or *Hdac2*, or the inhibition of HDAC activities with different inhibitors, prevents head regeneration [9]. Here, we show that the knockdown of *SoxB2*, or the inhibition of Class I and II HDACs prevent head regeneration by affecting the formation of a proliferative blastema.

In uninjured *Hydractinia* polyp, proliferative cells are primarily localized in the lower part of the body column [1] (Figure 1A). During head regeneration, a burst of cell proliferation occurs and the spatial distribution of proliferating cells changes to be concentrated at the blastema, where the new head will form; these events are necessary for head regeneration [1]. Because *SoxB2* knockdown inhibits head regeneration [9], we asked whether this phenotype is due to defects in cell proliferation. We decapitated animals and allowed them to regenerate in the presence of control or *SoxB2* double-stranded RNAs (dsRNAs). After 48 h, the animals were incubated with the mitotic marker EdU for 1 h, fixed and stained for EdU. In control animals we noticed a general increase in the number of EdU^+^ cells that were concentrated at the blastema, but in *SoxB2* RNAi animals we observed a general decrease in the number of EdU^+^ cells that stay concentrated in the lower part of body column. We observed the same phenotype using another mitotic marker, pospho-H3 (PH3) (Figure 1B). Hence, *SoxB2* knockdown compromises head regeneration by affecting cell proliferation. Our results are in agreement with the data showing that *SoxB2* knockdown decreases cell proliferation in intact polyps [9]. The general reduction in cell proliferation in *SoxB2* RNAi animals might be explained by the redistribution of *SoxB2*^+^ cells, that loose their normal restricted distribution pattern in the lower body column, becoming spread across the whole animal, including the blastema [9] (Figures 1A’, 1A’’, and C’).

**Figure 1.**
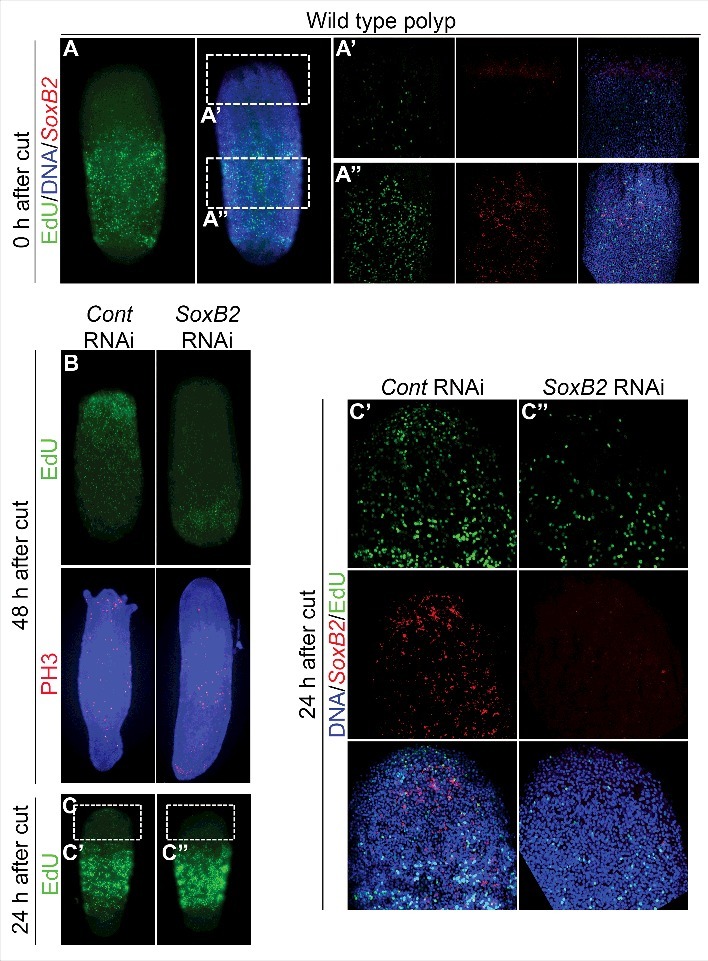
*SoxB2* knockdown prevents the formation of a proliferative blastema. (A, A’, A’’) EdU labeling and FISH showing the distribution of proliferating cells and *SoxB2^+^* cells in the body column of a polyp. (B) EdU and PH3 staining showing the distribution of proliferating cells in control and *SoxB2* RNAi animals. (C, C’, C’’) EdU pulse chase showing the migration of proliferating cells to the site of injury in control (C’) and *SoxB2* (C”) RNAi animals.

During head regeneration, mitotic cells migrate from the lower body part to the oral side to form a proliferative blastema [1]. To test if cell migration is affected upon *SoxB2* knockdown we performed EdU pulse chase experiments, by incubating animals in EdU for 1 hour before head amputation and treatment with control or *SoxB2* dsRNAs for 24 hs. Animals were then fixed and stained for EdU and *SoxB2* expression. In control animals high numbers of EdU^+^ and *SoxB2^+^* cells were detected in the developed blastema, but in *SoxB2* RNAi animals few EdU^+^ and *SoxB2^+^* cells were found in this area (Figures 1C, 1C’ and 1C’’). Hence, *SoxB2* downregulation inhibits head regeneration by also affecting cell migration. Previous work did show that the elimination of proliferating cells with gamma irradiation or mitomycin treatment inhibits blastema formation [1].

In injured *Hydractinia* polyps, a decline in HDAC activity, due to *Hdac2* knockdown or to HDAC activity inhibition, inhibits head regeneration [9] (Figures 2A and 2B). Here, similar to what was observed in *SoxB2* knockdown, we find that the inhibition of HDAC activity by TSA, a HDAC Class I and II inhibitor [19], induced defects in the formation of the proliferative blastema by affecting the migration of proliferating cells to the site of injury (Figures 2C-2E). Note that TSA treatment had no visible effect on cell proliferation (Figures 2A and 2B) [9]. The observed phenotype can, in part, be due to defects in *SoxB2* expression, because HDAC inhibition in regenerating animals induced a significant decrease in the expression level of *SoxB2* [9].

**Figure 2.**
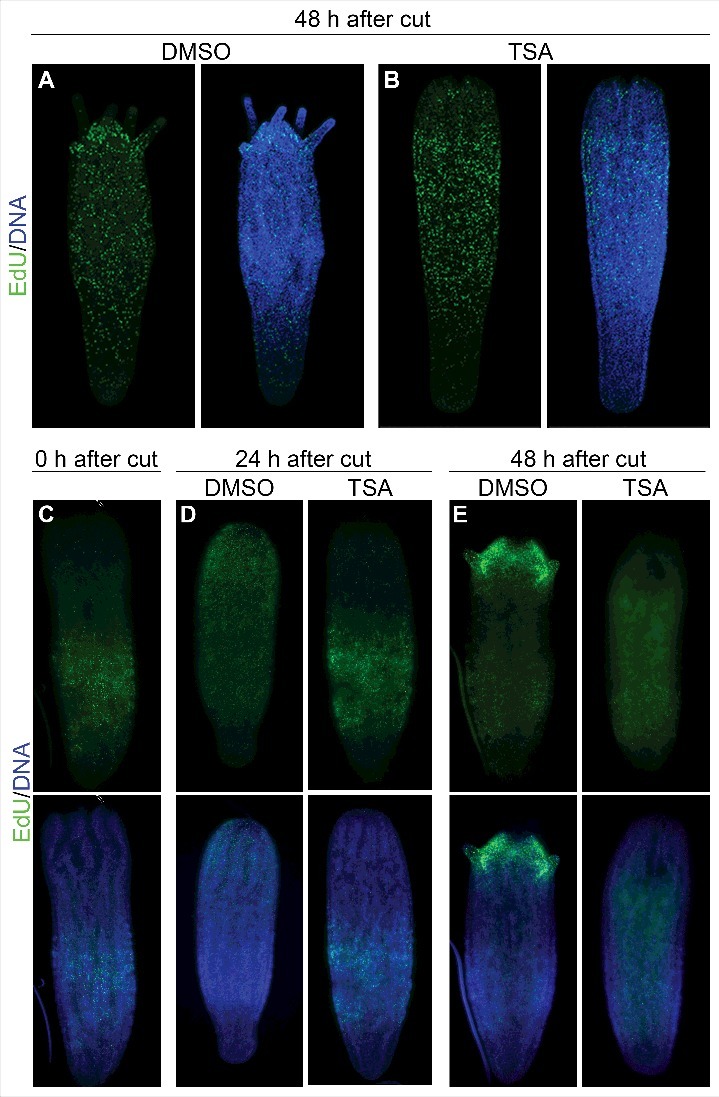
The inhibition of HDAC activity prevents the formation of a proliferative blastema. (A, B) EdU labeling showing the pattern of proliferating cells in regenerating polyps treated with DMSO or TSA. (C-E) EdU pulse chase showing that HDAC inhibition affects the migration of proliferating cells from the lower body part to the site of injury.

It was reported that Sox2 is involved in tracheal epithelium repair, spinal cord regeneration and transdifferentiation of support cells into hair cells during ear regeneration, in mice, *Xenopus* and zebrafish, respectively [12,13,15,16,20]. A number of studies have demonstrated that HDACs may contribute to the regeneration of many tissues, for example *Xenopus* tail and limb regeneration [17,18]. However, the underlying mechanism of action of SoxB and HDAC proteins in the regulation of regeneration remains unidentified. Here, we reported that SoxB2 and HDACs regulate head regeneration in *Hydractinia* by affecting the formation of a proliferative blastema. However, additional work is required to identify the mechanisms mediated by SoxB and HDAC proteins during regeneration.

## Material and methods

### Animals

Colonies of *Hydractinia echinata* were cultured in artificial seawater at 18**°**C under a 14/10 light/dark regime, and were fed five times a week with Artemia.

### EdU treatment and staining

Identification and pulse-chase of proliferating cells were performed as described in [1]. EdU staining was performed using Click-iT™ EdU Alexa Fluor™ [1] 488 Imaging Kit (C10337, ThermoFisher Scientific) according to the manufacturer's instructions.

### RNAi and drug treatment

dsRNA synthesis and treatment were performed as previously described [9].
